# SARS-CoV-2 Persistence and Cardiovascular Sequelae in the Post-COVID Era: A Public Health Microbiology Perspective on Sudden Cardiac Death and Pulmonary Thromboembolism

**DOI:** 10.3390/microorganisms14061256

**Published:** 2026-06-02

**Authors:** Cris Virgiliu Precup, Diana-Maria Mateescu, Alexandra Enache, Camelia Liana Buhas, Camelia-Oana Muresan

**Affiliations:** 1 Department of Biology and Life Sciences, Faculty of Medicine, “Vasile Goldiș” Western University of Arad, 310025 Arad, Romania; precupcris@yahoo.com; 2 Doctoral School, Department of General Medicine, “Victor Babeș” University of Medicine and Pharmacy, Eftimie Murgu Square 2, 300041 Timișoara, Romania; diana.mateescu@umft.ro; 3 Department of Legal Medicine, Timișoara Institute of Legal Medicine, 300041 Timișoara, Romania; muresan.camelia@umft.ro; 4 Ethics and Human Identification Research Center, “Victor Babeș” University of Medicine and Pharmacy, Eftimie Murgu Square 2, 300041 Timișoara, Romania; 5 Discipline of Forensic Medicine, Bioethics, Deontology, and Medical Law, Department of Neuroscience, “Victor Babeș” University of Medicine and Pharmacy, Eftimie Murgu Square 2, 300041 Timișoara, Romania; 6 Department of Morphological Disciplines, Faculty of Medicine and Pharmacy, University of Oradea, 410087 Oradea, Romania

**Keywords:** SARS-CoV-2, post-acute sequelae of COVID-19, viral persistence, ACE2, endothelial dysfunction, thromboinflammation, sudden cardiac death, pulmonary thromboembolism, molecular autopsy, public health microbiology

## Abstract

Post-acute sequelae of SARS-CoV-2 infection (PASC) extend well beyond the acute respiratory phase, with accumulating virological evidence that SARS-CoV-2 RNA, viral antigens, and proteolytic fragments may persist in cardiovascular and other extrapulmonary tissues, although the extent to which such detection represents replication-competent reservoirs versus residual viral material with uncertain pathological relevance remains under active investigation. Sudden cardiac death (SCD) and fatal pulmonary thromboembolism (PTE) have emerged as forensically and epidemiologically significant outcomes in individuals with prior infection, situated at the intersection of microbiology, public health, and forensic medicine. To synthesize current evidence on the virological mechanisms by which SARS-CoV-2 may contribute to post-acute sudden cardiac death (SCD) and pulmonary thromboembolism (PTE), the population-level epidemiology of these outcomes, and their implications for public health surveillance and forensic practice, we conducted a narrative review of PubMed (MEDLINE), Scopus, and Web of Science Core Collection. The search covered publications from January 2020 to December 2025 and focused on SARS-CoV-2 cellular tropism and tissue persistence, immune-mediated and thromboinflammatory mechanisms, excess cardiovascular and thromboembolic mortality, and autopsy-based pathological findings. After de-duplication of 1837 initially identified records (412 duplicates removed) and screening of 1425 unique records, 78 studies were retained for final synthesis based on virological, epidemiological, and forensic relevance. SARS-CoV-2 enters cardiomyocytes, pericytes, and vascular endothelial cells through ACE2-dependent mechanisms, with cathepsin L compensating for the limited cardiac expression of TMPRSS2. Viral RNA and antigen have been detected in cardiovascular and other extrapulmonary tissues months after symptom onset in selected autopsy series, although persistent detection of viral components does not necessarily indicate ongoing productive infection or direct tissue injury. Endothelial dysfunction, neutrophil extracellular trap (NET) formation, complement activation, and persistent thromboinflammation have been proposed as plausible mechanistic substrates for arrhythmogenic remodelling and thromboembolic events, although definitive causal pathways remain incompletely understood. Population-based studies document persistent excess cardiovascular mortality across multiple jurisdictions, with hazard ratios for pulmonary embolism remaining elevated months after acute infection, particularly in unvaccinated individuals. Autopsy series identify mixed pathological patterns including focal lymphocytic infiltrates, microvascular thrombosis, contraction-band necrosis, and cardiomyocyte vacuolation, although fulminant lymphocytic myocarditis fulfilling Dallas criteria remains uncommon. A microbiology-informed framework uniting tissue-based viral detection, standardized cardiac and pulmonary sampling protocols, and prospective post-mortem registries is needed to better characterize the potential contribution of SARS-CoV-2 to post-acute cardiovascular mortality and to support cause-of-death certification, public health surveillance, and medicolegal practice in the post-pandemic era. Many of the proposed mechanisms remain under active investigation, and definitive causal relationships between viral persistence and adverse cardiovascular outcomes have not yet been conclusively established.

## 1. Introduction

The COVID-19 pandemic, caused by severe acute respiratory syndrome coronavirus 2 (SARS-CoV-2), has produced the most consequential global infectious disease event of the twenty-first century. By the end of 2024, more than 776 million confirmed cases and over 7 million directly attributable deaths had been reported to the World Health Organization, with substantially larger excess-mortality estimates from independent demographic analyses [1,2]. As the acute phase of the pandemic has receded, attention has shifted to the post-acute sequelae of SARS-CoV-2 infection (PASC), commonly referred to as long COVID, and to the persistent excess mortality observed across multiple jurisdictions [2,3]. For the sake of clarity, throughout this review the terms “PASC” and “long COVID” are used interchangeably to refer to the broad clinical entity of post-acute SARS-CoV-2 sequelae, with PASC preferred when emphasizing the biological/pathophysiological substrate and long COVID preferred when discussing the clinically oriented or patient-centred literature; both terms are understood to encompass conditions persisting beyond the acute phase of SARS-CoV-2 infection.

From a public health microbiology standpoint, the post-COVID era raises a distinctive set of questions. SARS-CoV-2 is a respiratory virus with broad tissue tropism whose principal entry receptor, angiotensin-converting enzyme 2 (ACE2), is expressed across the cardiovascular system, including in cardiomyocytes, pericytes, and vascular endothelial cells [4,5,6]. A growing body of evidence indicates that viral RNA, subgenomic RNA, and viral antigens persist in extrapulmonary tissues for prolonged periods after the resolution of acute symptomatic illness, with autopsy-based studies documenting tissue-resident SARS-CoV-2 RNA more than seven months after symptom onset [7,8]. These observations have contributed to a broader microbiological and immunological reframing of PASC, in which persistent viral material has been hypothesized to sustain chronic immune activation, endothelial dysfunction, and thromboinflammation [9,10]. It should be emphasized, however, that viral persistence is only one of several proposed mechanisms underlying long COVID. Additional contributors that remain under active investigation include immune dysregulation and autoimmunity, autonomic dysfunction, mitochondrial injury, chronic low-grade inflammation, residual endothelial damage acquired during the acute phase, and indirect healthcare-related effects; their relative contribution to delayed cardiovascular outcomes has not yet been definitively established.

Two outcomes have become particularly salient at the interface of post-COVID microbiology, public health, and forensic pathology: sudden cardiac death (SCD) and fatal pulmonary thromboembolism (PTE). Excess cardiovascular mortality has been documented in the United States, with an estimated 228,524 excess cardiovascular deaths between 2020 and 2022 reported in a national surveillance analysis based on CDC mortality data [3], and approximately 90,160 excess cardiovascular deaths in the parallel period reported using a different modelling approach [11]. Population-based studies further demonstrate sustained increases in pulmonary embolism (PE) and deep vein thrombosis (DVT) risk after acute COVID-19, including in vaccinated individuals [12,13]. Vaccination has substantially reduced acute COVID-19 severity [14] and attenuated, though not eliminated, the risk of long COVID after breakthrough infection [15]; nonetheless, cardiovascular sequelae have been documented up to two years post-infection in large cohort analyses [16]. The mechanistic literature further indicates that ACE2-augmented inflammatory signalling drives multi-organ failure during severe acute illness and may contribute to the persistence of post-acute injury through dysregulated renin–angiotensin axis activity [17]. Both SCD and PTE frequently occur outside hospital settings, often in individuals without a documented active infection at the time of death, and therefore preferentially come to the attention of public health surveillance systems and forensic pathology services [18,19].

The present narrative review takes a public health microbiology perspective on these phenomena. We synthesize current evidence on (i) the cellular and molecular mechanisms of SARS-CoV-2 cardiovascular tropism and tissue persistence; (ii) the immunological, endothelial, and coagulation pathways linking infection to delayed cardiac and thromboembolic events; (iii) the population-level epidemiology of post-COVID cardiovascular and thromboembolic mortality; and (iv) the implications for autopsy-based surveillance, cause-of-death certification, and public health policy. The aim is to provide a framework that links virological mechanisms to population-level outcomes and to identify the microbiological tools—including tissue-based RT-PCR, immunohistochemistry for viral antigens, and molecular autopsy—that may help address the public health burden of post-COVID cardiovascular mortality. Unlike previously published reviews on long COVID cardiovascular complications, which have predominantly focused on clinical phenotypes or isolated mechanistic pathways, the present synthesis explicitly integrates forensic pathology with public health microbiology, with the aim of clarifying how post-mortem investigation can inform both individual cause-of-death determination and population-level surveillance.

## 2. Materials and Methods

A narrative review of the literature was conducted by searching PubMed (MEDLINE), Scopus, and Web of Science Core Collection for publications dated 1 January 2020 to 31 December 2025. Although the review was designed as a narrative synthesis rather than a formal systematic review, structured elements (multiple databases, defined thematic blocks, and a flow diagram) were used to improve transparency. Boolean operators (AND, OR, NOT) were applied across the thematic blocks; representative search strings combined terms such as ((SARS-CoV-2 OR COVID-19) AND (viral persistence OR tissue reservoir) AND (cardiovascular OR myocardium OR endothelium)) and ((sudden cardiac death OR pulmonary embolism) AND (autopsy OR forensic) AND (COVID-19 OR post-acute sequelae)). Full database-specific search strings are available from the corresponding author upon reasonable request. The search strategy combined Medical Subject Headings (MeSH) and free-text terms organized in four thematic blocks: (a) virological terms (“SARS-CoV-2”, “ACE2”, “viral tropism”, “viral persistence”, “tissue reservoir”, “spike protein”, “nucleocapsid”); (b) immunopathological terms (“endothelial dysfunction”, “endotheliitis”, “NETosis”, “thromboinflammation”, “complement activation”, “antiphospholipid antibodies”); (c) clinical and pathological outcomes (“sudden cardiac death”, “myocarditis”, “pulmonary embolism”, “venous thromboembolism”, “deep vein thrombosis”, “long COVID”, “PASC”, “post-acute sequelae”); and (d) public health and forensic terms (“excess mortality”, “cardiovascular mortality”, “autopsy”, “forensic pathology”, “cause of death”, “medicolegal”). Additional records were identified through manual searching of bibliographies of included articles and through targeted searches on specific topics (molecular autopsy, post-mortem computed tomography angiography, microclots in long COVID).

**Figure 1:**
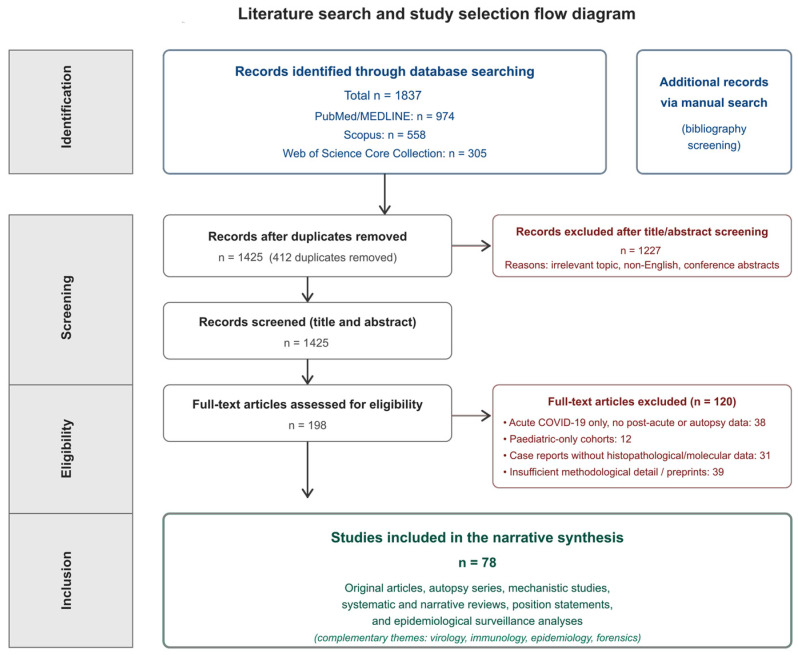
PRISMA-style flow diagram of study identification, screening, eligibility assessment, and inclusion.

Studies were eligible if they were original research articles, autopsy or biopsy case series, systematic or narrative reviews, or expert position statements addressing one or more of the four thematic blocks above. Both human and selected mechanistic in vitro/animal model studies were included where they provided foundational virological evidence directly relevant to human disease. Records were excluded if they were conference abstracts without full text, non-peer-reviewed preprints not subsequently published in peer-reviewed venues, paediatric-only cohorts (the focus of the present review being adult mortality), or studies addressing only acute COVID-19 without any post-acute, autopsy, or surveillance dimension. Non-English publications were excluded for operational reasons; this restriction is acknowledged as a potential source of language bias that may limit the international representativeness of the synthesis. Paediatric cohorts were excluded because post-COVID cardiovascular mortality in adults has a distinct epidemiology, pathophysiology, and forensic context compared with children, and paediatric long COVID warrants a dedicated review; consequently, the applicability of the present findings is restricted to adult populations. The literature search and screening process is summarized in Figure 1.

Title and abstract screening was performed by two authors (C.V.P. and D.-M.M.) independently, with disagreements resolved by discussion with a third author (A.E.); however, this review was not designed as a formal systematic review with PRISMA-guided dual-reviewer full-text screening or a structured risk-of-bias assessment, and the inclusion of both original studies and review articles in the final synthesis may have introduced some duplication in the interpretation of the same primary evidence base. These methodological limitations are acknowledged in Section 8. Study selection prioritized methodological rigour, virological and epidemiological relevance, and complementarity across the cardiovascular, thromboembolic, and forensic domains. Although no formal validated risk-of-bias instrument (such as ROBINS-I, Newcastle-Ottawa, or AMSTAR-2) was applied—consistent with the narrative design—each included study was qualitatively appraised against a set of pre-specified criteria covering: (i) study design and sample size relative to the question addressed; (ii) clarity and reproducibility of methods, including viral detection techniques (RT-PCR sensitivity, IHC validation, post-mortem interval reporting); (iii) appropriateness of controls and comparator groups; (iv) statistical handling of confounders, particularly in epidemiological cohort studies; and (v) consistency with independent replication where available. Studies meeting at least three of these criteria were classified as providing high-confidence evidence; those meeting one or two were retained as supportive evidence requiring cautious interpretation; and studies meeting none were excluded. This semi-quantitative appraisal is intended to provide transparency regarding the differential weight given to individual studies in the synthesis, while acknowledging that it falls short of a formal systematic-review-grade risk-of-bias assessment. The final synthesis incorporates 78 studies. The search strategy is summarized in Table 1.

## 3. SARS-CoV-2 Cardiovascular Tropism, Cellular Entry, and Tissue Persistence

A microbiological understanding of post-COVID cardiovascular mortality begins with the cellular and molecular mechanisms by which SARS-CoV-2 infects cardiovascular tissues and establishes long-lived reservoirs. The virus enters host cells through binding of the receptor-binding domain (RBD) of its spike (S) glycoprotein to ACE2, followed by S-protein priming by host proteases that expose the fusion peptide and enable membrane fusion [4]. While the canonical priming protease in respiratory epithelium is transmembrane protease serine 2 (TMPRSS2), the cardiovascular system displays a distinct proteolytic landscape that has direct implications for viral tropism, persistence, and post-acute pathology.

### 3.1. ACE2 Distribution and Cardiovascular Cellular Tropism

Single-cell transcriptomic analyses of healthy and diseased adult human heart consistently show that ACE2 is most abundantly expressed in pericytes, followed by fibroblasts and cardiomyocytes, with lower expression in vascular endothelial cells [5,6,20]. ACE2 expression is upregulated in failing hearts and in patients with pre-existing cardiovascular disease, providing a plausible molecular substrate for the disproportionate impact of SARS-CoV-2 on individuals with cardiac comorbidities [6,20]. Critically, ACE2 and TMPRSS2 are essentially not co-expressed in cardiac cells, indicating that cardiac infection cannot proceed through the canonical respiratory entry pathway [20,21].

In place of TMPRSS2, cardiac cells appear to rely on cathepsin L, a lysosomal cysteine protease that cleaves S protein after endocytic uptake and that is highly expressed in cardiomyocytes [20,21]. This alternative entry route—termed the endosomal pathway—has been confirmed in studies using human pluripotent stem cell-derived cardiomyocytes (hPSC-CMs), where SARS-CoV-2 infection is highly productive: viral transcripts accounted for approximately 88% of total mRNA in hiPSC-CMs in one report, with prolific particle release through smooth-walled exocytic vesicles and surface budding [21,22]. Infection is accompanied by disruption of the contractile cytoskeleton, electrical and mechanical dysfunction, and progressive cell death [22]. These findings provide mechanistic evidence that SARS-CoV-2 can infect cardiac cells at the cellular level through an entry pathway distinct from the canonical respiratory route. Pharmacological studies of intracellular antiviral activity have further highlighted the importance of cellular and biochemical context for predicting in vitro and in vivo anti-SARS-CoV-2 activity, with implications for the design of agents targeting cardiac and other extrapulmonary compartments [23]. The extent to which these in vitro and stem-cell-derived observations translate to sustained, productive cardiotropism in vivo, particularly in the post-acute setting, remains to be fully established and should be confirmed by additional human autopsy and tissue-level studies.

**Figure 2:**
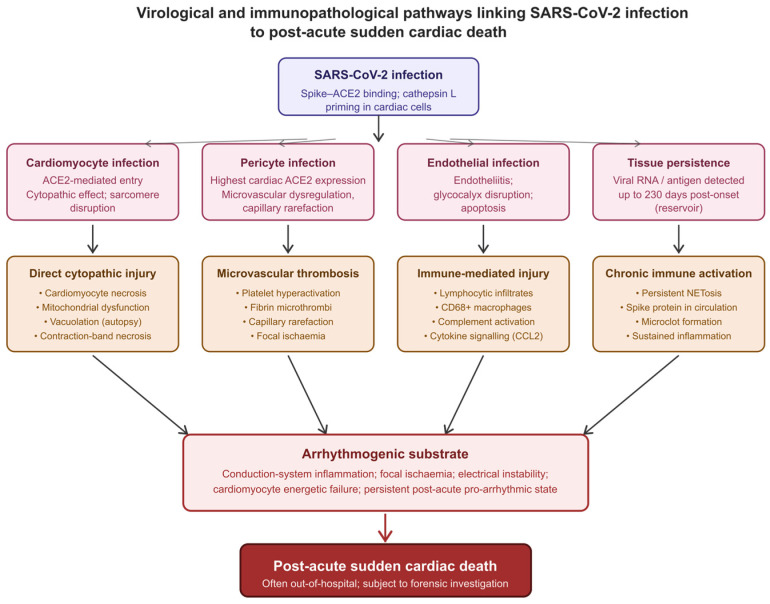
Proposed mechanisms linking SARS-CoV-2 cardiovascular tropism to microvascular dysfunction and arrhythmogenic remodeling. SARS-CoV-2 infects cardiomyocytes, pericytes, and vascular endothelial cells through ACE2-dependent mechanisms, with cathepsin L facilitating endosomal entry in cardiac tissue. Pericyte dysfunction and endothelial injury contribute to capillary instability, microvascular rarefaction, endothelial activation, thromboinflammation, and impaired myocardial perfusion. Persistent immune activation, NET formation, complement activation, and microthrombus formation promote myocardial injury, fibrosis, electrical instability, and increased susceptibility to arrhythmias and sudden cardiac death.

Vascular endothelial cells, although expressing lower levels of ACE2 than pericytes, are nonetheless infectable by SARS-CoV-2 in vitro and in vivo, with endothelial infection demonstrated in autopsy tissues from multiple organs [24,25]. Pericyte infection has been proposed as a particularly consequential event for cardiac microcirculation, given the role of pericytes in capillary integrity, blood–brain and blood–myocardial barriers, and microvascular tone regulation [5,26]. Loss or dysfunction of cardiac pericytes following SARS-CoV-2 infection may contribute to microvascular rarefaction, impaired perfusion, and a substrate for arrhythmogenesis, as shown in Figure 2.

### 3.2. Tissue Persistence and Viral Reservoirs in the Post-Acute Phase

The most consequential virological development of the past three years has been the recognition that SARS-CoV-2 is not consistently cleared at the resolution of acute illness. In a landmark NIH autopsy study of 44 patients, Stein and colleagues performed comprehensive sampling of up to 85 distinct anatomic locations per case and detected SARS-CoV-2 RNA in 79 of these sites across the cohort, including cardiovascular and brain tissues, for up to 230 days following symptom onset; subgenomic RNA (a marker of active viral replication) was detected at extrapulmonary sites in a subset of cases [7]. These findings established that SARS-CoV-2 nucleic acids can be widely distributed beyond the respiratory tract early in infection and that viral genetic material may persist in extrapulmonary tissues across timescales potentially relevant to PASC. It should be noted, however, that the cohort consisted of patients who died during or shortly after severe acute infection, that detection of viral RNA does not by itself establish ongoing productive replication, and that the post-mortem interval, fixation procedures, and RT-PCR sensitivity can substantially influence reported detection rates across studies.

Persistent viral material has subsequently been documented in multiple anatomic compartments using complementary techniques. Spike (S) protein has been detected in plasma of patients with PASC up to 12 months after acute illness, predominantly in the unbound form, suggesting either slow tissue release from reservoirs or local production in distant compartments [27]. Nucleocapsid (N) antigenaemia has similarly been documented in patients with persistent post-COVID symptoms, with circulating viral antigen detected months after acute infection in association with PASC phenotypes [28]. Viral antigen has also been demonstrated in tonsillar and adenoid tissue from children with long COVID [29] and in intestinal biopsies from approximately half of long COVID patients sampled four months after acute infection [30]. Tissue-resident viral RNA has also been demonstrated at the skull–meninges–brain axis up to 28 days post-infection in murine models and in human autopsy tissues, persisting after pulmonary clearance [31].

The cardiovascular implications of these reservoir findings are substantial. Persistent viral antigen, even in the absence of replication-competent virus, can sustain local immune activation, endothelial dysfunction, and pro-thrombotic signalling [9,32]. Spike protein in circulation has been shown to interfere directly with the coagulation cascade through competitive binding to heparan sulfate, induce microthrombus formation, and contribute to fibrin deposition with abnormal amyloid-like architecture (so-called “microclots”) that resist normal fibrinolysis [33,34]. Recent work has further demonstrated that proteolytic fragments of SARS-CoV-2 proteins can self-assemble into immunomimetic supramolecular complexes that drive sustained inflammation independently of intact viral replication [35]. These mechanisms have been proposed to provide a microbiological substrate for the chronic thromboinflammatory state observed in a subset of patients with PASC, and may contribute to the elevated risk of cardiovascular and thromboembolic events documented months after acute infection. For balance, it should be noted that not all studies have identified persistent viral antigens or nucleic acids in patients with long COVID, that the proportion of PASC patients with detectable tissue or circulating viral material varies widely across cohorts, and that the pathological significance of viral protein detected at trace levels (in the absence of accompanying inflammatory infiltrate or evidence of replication) remains debated.

### 3.3. Direct Cardiac Infection at Autopsy: Frequency and Interpretation

Cardiac SARS-CoV-2 detection at autopsy varies markedly across studies, reflecting differences in patient selection, post-mortem interval, sampling protocols, and detection techniques. In a study of 39 consecutive autopsy cases from Hamburg by Lindner and colleagues, SARS-CoV-2 RNA was detected in cardiac tissue in 24 of 39 cases (61.5%), with viral loads exceeding 1000 copies per microgram of RNA in 16 of 39 (41.0%) [36]. Importantly, even at high viral loads, this study did not identify lymphocytic myocarditis fulfilling Dallas criteria—defined as inflammatory infiltrate of the myocardium associated with adjacent myocyte injury and necrosis not characteristic of an ischaemic event—although a pro-inflammatory cytokine signature was detected in tissues with high viral burden, illustrating that cardiac viral presence and classical histological myocarditis are partially decoupled [36].

In a multicentre cardiovascular pathology study, Basso and colleagues examined 21 hearts from COVID-19 decedents and identified myocarditis (defined as inflammatory infiltrate associated with myocyte injury in multiple foci) in 3 of 21 cases (14.3%), with additional non-myocarditis findings including focal lymphocytic infiltrates, contraction-band necrosis, and microvascular thrombi [37]. SARS-CoV-2 RNA and protein were detected by RT-PCR and immunohistochemistry in a subset of these cases, frequently in interstitial macrophages rather than in cardiomyocytes themselves [37]. A subsequent systematic literature review of 277 autopsied hearts across 22 publications estimated that histologically defined myocarditis was present in approximately 7.2% of cases, and likely in less than 2% of cases when stricter functional criteria were applied [38]. The European Society of Cardiology Working Group on Myocardial and Pericardial Diseases has emphasized that the histopathological diagnosis of myocarditis requires not only inflammatory infiltrates but evidence of myocyte injury, and that the detection of viral genome alone—in the absence of accompanying histological criteria—is insufficient to establish a diagnosis of viral myocarditis [39].

These data support a nuanced microbiological interpretation. Cardiac SARS-CoV-2 detection has been frequently reported in deaths occurring during the acute phase of infection, but does not regularly produce fulminant lymphocytic myocarditis. The frequency of viral detection across autopsy series varies markedly and is sensitive to differences in RT-PCR sensitivity, tissue sampling, fixation, and post-mortem interval; incidental viral RNA detection without accompanying pathological correlates should not be over-interpreted as evidence of causal contribution. Importantly, most published autopsy series concern patients who died during acute or severe COVID-19 rather than exclusively in the post-acute, long-COVID setting; extrapolation of acute-phase pathological findings to delayed sudden cardiac death therefore requires caution. Instead, the dominant cardiac patterns are mixed: microvascular thrombosis, focal interstitial inflammation, macrophage-predominant infiltrates, and cardiomyocyte injury that may or may not be associated with detectable viral RNA. From a public health surveillance perspective, this argues against using “myocarditis” as a binary diagnostic category in post-COVID cardiac death investigation, and in favour of standardized, multimodal protocols incorporating tissue-based viral detection, histopathology, and immunohistochemistry [40]. It should also be emphasized that myocardial inflammation, microvascular thrombosis, and endothelial activation are not unique to SARS-CoV-2 and may be observed in other severe viral infections (notably influenza A) and in systemic inflammatory states; this contextual specificity should be considered when ascribing observed pathological patterns to SARS-CoV-2 in the absence of direct virological correlation.

## 4. Endothelial Dysfunction, Thromboinflammation, and the Coagulation Disturbance of PASC

The cardiovascular consequences of SARS-CoV-2 infection extend beyond direct cellular cytopathic effects. A central pathophysiological feature of both acute COVID-19 and PASC is a thromboinflammatory state in which the vascular endothelium, the innate immune system, and the coagulation cascade are simultaneously and persistently dysregulated. From a public health microbiology perspective, this state provides the mechanistic bridge between virological events at the cellular level and the population-level excess of cardiovascular and thromboembolic mortality.

### 4.1. Endothelial Infection and Endotheliitis

In an early autopsy study by Varga and colleagues, SARS-CoV-2 was demonstrated within vascular endothelial cells of multiple organs, accompanied by accumulation of inflammatory cells in the endothelium (“endotheliitis”) and apoptotic body formation [24]. Subsequent work by Ackermann and colleagues, comparing pulmonary specimens from patients with COVID-19 and influenza A (H1N1), identified distinctive vascular features in COVID-19, including widespread endothelial injury associated with intracellular SARS-CoV-2 virions, severe endothelial damage with disruption of cell membranes, alveolar capillary microthrombi nine times more prevalent than in influenza, and significant new vessel growth (intussusceptive angiogenesis) [25]. These findings positioned endothelial infection as a defining feature of COVID-19 pathology that distinguishes it from other respiratory viral infections.

Many of the endothelial findings described in the autopsy literature originate from patients with severe acute COVID-19, and the extent to which these acute-phase changes persist into the post-acute, long-COVID setting requires careful distinction. Following acute infection, endothelial dysfunction does not consistently resolve in all patients. Functional studies in patients with persistent post-COVID symptoms have demonstrated impaired flow-mediated dilatation and elevated circulating markers of endothelial activation—including von Willebrand factor (VWF), soluble vascular cell adhesion molecule-1 (sVCAM-1), and angiopoietin-2—months after acute illness [41,42]. Endothelial dysfunction in PASC correlates with reduced exercise tolerance and persistent inflammation and is associated with elevated thrombin generation capacity and altered fibrinolysis [10,43]. CCL2-mediated endothelial injury has been mechanistically linked to cardiomyocyte dysfunction in long COVID through endothelium–cardiomyocyte crosstalk in murine and stem-cell-derived models, identifying a candidate pathway for delayed cardiac dysfunction in the absence of overt myocarditis [44]. Structural studies of the endothelial glycocalyx and its interactions with circulating lipoproteins have further clarified how disruption of this critical vascular interface may compound endothelial injury and contribute to persistent microvascular dysfunction following infection [45].

### 4.2. NETosis, Platelet Hyperactivation, and Complement Activation

Neutrophil extracellular trap (NET) formation, or NETosis, is a defining innate immune response to SARS-CoV-2 and a central driver of immunothrombosis. NETs are extracellular DNA–histone scaffolds decorated with neutrophil-derived antimicrobial proteins; they trap pathogens and also promote thrombus formation by activating platelets and the contact phase of coagulation [46]. In COVID-19, circulating markers of NETosis (cell-free DNA, citrullinated histone H3, myeloperoxidase–DNA complexes) are elevated during acute illness and have been shown to remain elevated in PASC, providing a sustained pro-thrombotic stimulus [46,47].

Platelet hyperactivation accompanies NETosis in both acute COVID-19 and PASC. Studies of patients with PASC have demonstrated increased platelet aggregation, elevated soluble P-selectin, and dysregulated platelet transcriptomic signatures consistent with a chronic activation state [48]. Importantly, exposure of platelets to SARS-CoV-2 spike protein in vitro is sufficient to induce activation through ACE2-dependent signalling, suggesting that circulating spike from tissue reservoirs may directly contribute to the prothrombotic phenotype of long COVID [49]. Recent mechanistic work identifying novel intracellular regulators of platelet activation and thrombus formation, such as the orphan nuclear receptor NR4A1, further highlights the complexity of platelet biology in thromboinflammatory states and may inform future research into the persistent platelet dysregulation observed in PASC [50].

Complement activation provides a third arm of the thromboinflammatory triad. The lectin and alternative complement pathways are activated by SARS-CoV-2 spike, and terminal complement complex (C5b-9) deposition has been documented at sites of microvascular injury in lung, kidney, and skin in COVID-19 [51]. Complement activation amplifies endothelial injury, promotes NET formation, and enhances tissue factor expression on monocytes—closing a self-reinforcing loop with NETosis platelet activation, which preserves the thromboinflammatory state during PASC [47,51].

### 4.3. Persistent Coagulopathy and Microclot Formation

The coagulation disturbance of acute COVID-19—characterized by elevated D-dimer, fibrinogen, factor VIII, and VWF, with relative suppression of natural anticoagulants—has been shown to persist in a substantial subset of patients with PASC [10,34,52]. Thromboelastography studies in critically ill COVID-19 patients have documented hypercoagulable profiles characterized by shortened reaction times and increased clot strength, reflecting both increased fibrinogen and platelet hyperactivity [53]. The persistence of these abnormalities into the post-acute phase, although attenuated, has been described as part of the broader dysregulated host response that characterizes long COVID syndromes [54]. Pretorius and colleagues have reported the presence of fibrinaloid “microclots”—fibrin polymers with abnormal amyloid-like architecture that are reported to be resistant to standard fibrinolysis—in the plasma of patients with long COVID [34,52]. It should be noted that the microclot hypothesis remains an area of active scientific debate: independent replication has been limited, the detection methodology has been criticized as methodologically heterogeneous, and several groups have not consistently observed the same fibrin morphology in independent long-COVID cohorts. These observations should therefore be regarded as preliminary, and their pathological significance as the subject of ongoing investigation. These microclots can entrap inflammatory and pro-thrombotic molecules and have been proposed as both a marker and a mechanistic substrate of PASC. Spike protein has been identified within microclot structures, supporting a direct virological role in their formation [33,52].

A further coagulation perturbation specific to SARS-CoV-2 is the de novo induction of antiphospholipid antibodies (aPL), including lupus anticoagulant, anticardiolipin, and anti-β2-glycoprotein I antibodies [55]. While the persistence and clinical relevance of these autoantibodies after acute infection remain debated, their detection in patients with thromboembolic events after COVID-19 is forensically and clinically relevant. Inherited thrombophilias (factor V Leiden, prothrombin gene G20210A variant) further amplify post-COVID thromboembolic risk, although their interaction with SARS-CoV-2-induced coagulopathy is incompletely characterized [13].

### 4.4. Mitochondrial Dysfunction and Cardiomyocyte Stress

Mitochondrial injury represents an additional, underrecognized mechanism of post-COVID cardiac dysfunction. Endomyocardial biopsy analysis from patients with long COVID-associated cardiovascular manifestations has demonstrated extensive mitochondrial vacuolation, myofilament degradation, and lipofuscin accumulation in cardiomyocytes by electron microscopy [56]. SARS-CoV-2 ORF proteins have been shown to localize to mitochondria, disrupt mitochondrial membrane potential, and impair oxidative phosphorylation in infected cells—providing a molecular substrate for the cardiomyocyte vacuolation pattern reported in autopsy series of post-COVID sudden cardiac death [56,57]. Mitochondrial dysfunction generates reactive oxygen species, sensitizes cardiomyocytes to arrhythmia, and may contribute to the focal contraction-band necrosis identified in some post-COVID autopsy cases [57]. The mechanistic landscape underlying post-COVID cardiovascular sequelae is summarized in Figure 2. It is important to distinguish, within this landscape, between mechanisms supported by convergent evidence across independent studies and those that remain hypothesis-generating. Endothelial injury and dysfunction, neutrophil extracellular trap formation, platelet hyperactivation, and acute-phase complement activation are established features of severe COVID-19 supported by multiple independent autopsy series, in vitro studies, and clinical cohorts. By contrast, the pathological significance of fibrinaloid microclots, the role of immunomimetic supramolecular peptide complexes in driving sustained inflammation, and the direct causal contribution of mitochondrial damage to long-term cardiac arrhythmogenesis in PASC remain emerging hypotheses, supported predominantly by single-group findings or limited replication, and should be regarded as scientifically promising but not yet conclusively established. Figure 2 and the accompanying tables should be read with this distinction in mind.

## 5. Population-Level Epidemiology of Post-COVID Cardiovascular and Thromboembolic Mortality

Translating virological mechanisms into public health impact requires population-level data on excess mortality. Multiple independent surveillance analyses now provide convergent evidence that the COVID-19 pandemic has produced a substantial and partially persistent excess of cardiovascular and thromboembolic deaths.

### 5.1. Excess Cardiovascular Mortality

In a CDC-based analysis of national vital statistics, Woodruff and colleagues estimated that 228,524 excess cardiovascular deaths (95% CI: 199,980–257,190) occurred in the United States from 2020 to 2022, representing a 9.0% (95% CI: 7.8–10.3) excess relative to pre-2020 trends [3]. The age-adjusted cardiovascular mortality rate, which had declined by 8.9% from 2010 to 2019, increased by 9.3% from 2019 to 2022—effectively erasing nearly a decade of gains. Excess deaths were highest in adults aged 35–54 years (13.5%), Black adults (10.6%), and Asian or Pacific Islander adults (12.2%), highlighting demographic disparities in the cardiovascular impact of the pandemic [3].

Using a different methodology—temporal modelling of expected versus observed cardiovascular deaths against pandemic waves—Han and colleagues estimated 90,160 excess cardiovascular deaths in the United States from March 2020 to March 2022 (4.9% above expected), with the largest excesses coinciding with peaks of COVID-19 deaths in March–June 2020 and June–November 2021 [11]. The differences between these estimates reflect different modelling approaches and time windows rather than contradictory findings; collectively they document a robust signal of excess cardiovascular mortality during the pandemic. The interpretation of this excess, however, requires considerable caution. Multiple non-virological factors are likely to have contributed concurrently, including disrupted access to acute and chronic cardiovascular care, overwhelmed emergency medical systems, deferred elective procedures, interrupted chronic disease management, reduced help-seeking behaviour during pandemic peaks, socioeconomic stress, and indirect effects of public health restrictions. These factors may influence cardiovascular mortality trends independently of direct SARS-CoV-2 effects, and most cited observational studies demonstrate association rather than establishing causal contribution of viral persistence to cardiovascular outcomes. In addition, much of the population-level evidence is concentrated in North America and Europe; additional data from Asian, African, and Latin American settings would improve the international generalizability of these findings. A 2025 update found that, while the pandemic-era excess began to attenuate after 2022, age-adjusted cardiac mortality remained above pre-pandemic projections through 2024, particularly in elderly subgroups [58].

Long-term cohort data from the U.S. Department of Veterans Affairs further demonstrate that individuals with prior SARS-CoV-2 infection face elevated 12-month risks for a wide range of cardiovascular outcomes—cerebrovascular disease, dysrhythmias, ischemic heart disease, myocarditis, heart failure, and thromboembolic disease—even after non-hospitalized acute illness [59]. The hazard ratios are graded by acute severity (highest in those hospitalized or admitted to intensive care) but remain elevated for non-hospitalized individuals, indicating that the cardiovascular risk of SARS-CoV-2 is not confined to severe acute disease.

### 5.2. Pulmonary Embolism and Deep Vein Thrombosis

Population-based studies provide consistent evidence that COVID-19 is associated with markedly elevated risks of PE and DVT during the acute and post-acute phases. In a Korean nationwide cohort study of 1,601,835 individuals with COVID-19 matched to 14,011,285 uninfected controls, Kim and colleagues found that COVID-19 was associated with a 6.25-fold increased risk of PE (adjusted hazard ratio [aHR] 6.25; 95% CI 3.67–10.66) and a 3.05-fold increased risk of DVT (aHR 3.05; 95% CI 1.75–5.29) in unvaccinated individuals, and that residual elevated PE risk persisted in vaccinated individuals (aHR 1.48; 95% CI 1.15–1.88) [12]. A Swedish population-based cohort similarly demonstrated elevated long-term PE and DVT risk after COVID-19, with significantly increased hazard ratios for PE in the 60–180-day window after acute infection and continuing—although attenuated—beyond 180 days [13]. Table 2 summarizes hazard ratios, follow-up durations, and key outcomes across major population-level studies of post-COVID cardiovascular and thromboembolic risk.

These population-level signals are particularly relevant for forensic and public health practice because PE deaths are systematically under-ascertained in routine death certificate data. A 10-year retrospective forensic autopsy study from northern and western Denmark reported that PTE was the cause of death in 1.6% of forensic autopsies but in only 0.3% of cause-of-death registry entries, a nearly six-fold discrepancy [60]. Out-of-hospital deaths, in particular, are at risk of misclassification in the absence of autopsy, raising concerns that the population-level burden of post-COVID PTE is underestimated by routine surveillance.

### 5.3. Out-of-Hospital Cardiac Arrest and Sudden Cardiac Death

The epidemiology of out-of-hospital cardiac arrest (OHCA) shifted measurably during the pandemic. National and regional studies documented increased OHCA incidence and reduced survival to discharge during pandemic waves, reflecting both direct COVID-19 effects and indirect factors such as delayed presentation and disrupted emergency services [61]. Importantly, the incidence of OHCA did not return to pre-pandemic baselines in 2022–2023, and forensic data suggest that a non-trivial fraction of post-pandemic OHCA cases involve individuals with prior, sometimes asymptomatic, SARS-CoV-2 infection [61,62]. Among individuals younger than 50 years without classical cardiac risk factors, post-COVID SCD has been increasingly identified at autopsy, raising the public health priority of systematic post-mortem investigation in this group [62].

The shift in cardiac death from in-hospital to out-of-hospital settings observed during and after the pandemic [61] increases the proportion of cardiac deaths that come to forensic attention, and therefore the importance of standardized post-mortem virological evaluation. Without such evaluation, the contribution of SARS-CoV-2 to post-acute SCD will remain a microbiological “blind spot” in mortality surveillance.

### 5.4. Modifying Effect of Vaccination and Breakthrough Infection

Vaccination against SARS-CoV-2 has substantially altered the epidemiology of both acute and post-acute cardiovascular outcomes, and any contemporary assessment of post-COVID cardiovascular mortality must take this modifying effect into account. mRNA and adenoviral-vector vaccines have demonstrated high efficacy in reducing severe acute COVID-19 and death [14], and large cohort and population-based studies have consistently shown that pre-infection vaccination is associated with reduced—though not abolished—risk of subsequent long COVID symptoms and cardiovascular sequelae after breakthrough infection [15]. In the nationwide Korean cohort discussed above, the risk of pulmonary embolism was substantially attenuated in vaccinated compared with unvaccinated individuals (aHR 1.48; 95% CI 1.15–1.88 versus 6.25; 95% CI 3.67–10.66, respectively), although a residual elevated risk persisted [12]. Veterans Affairs cohort analyses have similarly documented attenuated but not eliminated cardiovascular risk after breakthrough infection in vaccinated individuals [15,16]. The mechanisms underlying this protective effect are likely multifactorial and include reduced viral replication, lower peak viral load, attenuated systemic inflammatory response, and probably reduced establishment of extrapulmonary tissue viral material. By contrast, separate and distinct safety signals—most notably a small absolute excess of acute, predominantly self-limiting myocarditis and pericarditis in young males following mRNA vaccination—have been characterized in pharmacovigilance surveillance and are largely temporally and pathologically distinct from the post-infection cardiovascular sequelae discussed in this review. From a forensic-microbiology standpoint, vaccination status at the time of acute infection should be recorded in post-mortem investigation protocols, and analyses of post-COVID cardiovascular mortality should ideally stratify outcomes by vaccination status, variant era, and time since acute infection to capture the heterogeneity of contemporary post-acute risk.

## 6. Post-Mortem Investigation of Post-COVID Cardiovascular Deaths

The microbiological and pathological investigation of suspected post-COVID cardiovascular deaths requires integration of standard autopsy practice with virological and molecular techniques. The aim is twofold: to establish whether SARS-CoV-2 contributed causally to the terminal event, and to generate population-level evidence on the contribution of the virus to post-acute mortality.

### 6.1. Sudden Cardiac Death: Autopsy Findings and Microbiological Workup

Sudden cardiac death is defined as unexpected natural death from a cardiac cause within one hour of symptom onset in a person without any prior condition that would appear fatal [63]. Autopsy investigation of suspected post-COVID SCD requires extensive histopathological sampling of the myocardium and dedicated examination of the conduction system. Sampling from at least six anatomical regions—including the right and left ventricular free walls, interventricular septum, sinoatrial node, atrioventricular node, and bundle of His—is recommended by the Association for European Cardiovascular Pathology [64,65].

Reported autopsy findings in COVID-19-related cardiac deaths are heterogeneous. Across multiple series, the dominant patterns include focal lymphocytic infiltrates that frequently fall short of Dallas criteria for myocarditis, contraction-band necrosis, microvascular thrombi, cardiomyocyte vacuolation with reduced staining intensity on histochemical stains, and interstitial fibrosis [36,37,38,66,67]. Comprehensive narrative reviews of the literature on myocarditis and myocardial injury in long COVID syndromes have similarly emphasized this heterogeneous and predominantly non-fulminant histopathological pattern [68]. Detailed pathological characterization has further demonstrated that nonocclusive fibrin microthrombi within small intramyocardial vessels—often without accompanying inflammatory infiltrate—constitute a distinctive and clinically underrecognized pattern of cardiac injury in COVID-19 [69]. Table 3 summarizes representative autopsy findings from published series of COVID-19-related cardiac deaths.

A microbiological workup is essential to discriminate cardiac SARS-CoV-2 involvement from incidental detection. Recommended elements include (i) RT-PCR for SARS-CoV-2 RNA on formalin-fixed paraffin-embedded (FFPE) cardiac tissue blocks, with quantification of viral load to distinguish high-burden from trace detection; (ii) immunohistochemistry for SARS-CoV-2 nucleocapsid and spike antigens to localize viral protein to specific cell types (cardiomyocytes, pericytes, endothelial cells, interstitial macrophages); (iii) detection of subgenomic RNA where feasible, as a marker of active viral replication versus residual genomic RNA; and (iv) consideration of next-generation sequencing for viral variant identification, particularly in cases where the timing of acute infection is uncertain [7,40,70]. Complementary rapid molecular platforms—including CRISPR/Cas12a-based surface plasmon resonance assays developed for the specific diagnosis of SARS-CoV-2 variants such as Omicron—illustrate the expanding toolkit available for variant-resolved viral detection that may, in future, be adapted to forensic and post-mortem tissue applications [71]. Detection of SARS-CoV-2 RNA or antigen in cardiac tissue, particularly when accompanied by appropriate histological correlates, can support a probabilistic causal association even when nasopharyngeal swabs are negative post mortem [70]. Conversely, isolated detection of viral RNA in the absence of compatible histopathology, or against a background of high post-mortem interval and tissue degradation, should be interpreted cautiously and not taken in isolation as definitive evidence of causality in sudden cardiac death; false-negative RT-PCR results due to tissue degradation, the potential for low-level contamination in molecular workflows, and the limitations of immunohistochemistry (including antibody specificity and antigen retrieval variability) must all be considered, and findings should always be integrated with clinical history and the exclusion of alternative causes of death.

In cases where comprehensive autopsy fails to identify a structural cause of death—affecting up to 30% of sudden unexplained deaths in young individuals—molecular autopsy (post-mortem genetic analysis of arrhythmia-susceptibility genes) should be considered to evaluate inherited channelopathies and cardiomyopathies, which may unmask in the setting of SARS-CoV-2-induced cardiomyocyte stress [72].

### 6.2. Pulmonary Thromboembolism: Autopsy Investigation and Differentiation from Post-Mortem Clots

Forensic autopsy of suspected fatal PTE requires systematic dissection of the pulmonary vasculature—opening the main pulmonary trunk and tracing primary, lobar, segmental, and subsegmental branches—together with examination of the deep veins of the lower extremities, pelvic veins, and inferior vena cava [60,73]. A critical microbiological–pathological prerequisite is the differentiation of true ante-mortem thrombi from post-mortem cadaveric clots; the latter lack the layered fibrin architecture of true ante-mortem thrombi and show no signs of organization [74]. Acutely formed ante-mortem thrombi exhibit a laminated pattern of fibrin and red blood cells without organization, while progressive organization over days to weeks is characterized by ingrowth of endothelial cells and fibroblasts, eventually leading to partial or complete recanalization [73]. Histological dating of pulmonary thrombi can clarify whether the fatal event was a new acute episode superimposed on an organized chronic thrombus, with implications for both clinical history reconstruction and medicolegal analysis.

Post-mortem computed tomography pulmonary angiography (PMCTA) is an increasingly available adjunct that can demonstrate filling defects in the pulmonary vasculature and right-heart distension prior to autopsy dissection, with sensitivity comparable to autopsy in selected studies [75]. Combined with histological thrombus dating and immunohistochemical assessment of pulmonary endothelium for SARS-CoV-2 antigens, PMCTA enables a more comprehensive characterization of the post-COVID PTE phenotype than dissection alone.

Risk factors for fatal PTE in post-COVID individuals include classical thrombosis risk factors (advanced age, immobilization, prior venous thromboembolism, obesity, active malignancy, inherited thrombophilia) amplified by SARS-CoV-2-specific mechanisms (endotheliitis, NETosis, antiphospholipid antibody induction, persistent thromboinflammation) [13,47,55]. A distinctive pattern in post-COVID PTE is the relative frequency of in situ pulmonary thrombosis without an identifiable peripheral DVT source—consistent with primary pulmonary endothelial injury and microthrombosis driven by the virus itself [25,76]. Comprehensive autopsy experiences from large urban centres in the United States [77] and Germany [78] have similarly documented mixed patterns of acute pulmonary embolism, alveolar microthrombi, and diffuse alveolar damage, with venous thromboembolic events identified as a common cause of death in COVID-19 inpatients in a prospective Hamburg autopsy cohort [79]. Table 4 summarizes risk factors for fatal PTE in the post-COVID setting.

### 6.3. A Proposed Microbiological–Pathological Protocol

**Figure 3:**
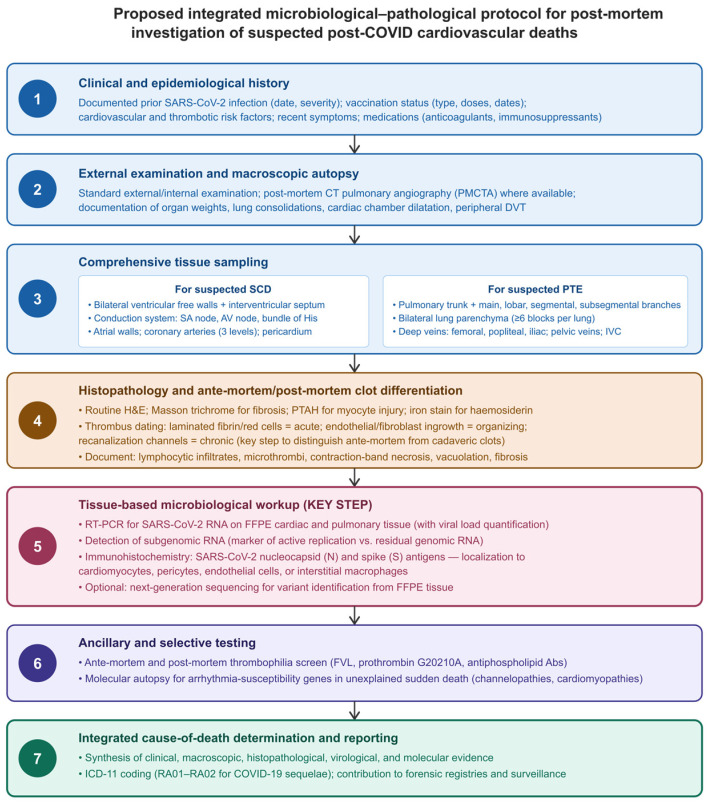
Proposed integrated forensic microbiology protocol for the investigation of post-COVID sudden cardiac death and pulmonary thromboembolism, combining autopsy, histopathology, tissue-based SARS-CoV-2 detection, thrombophilia assessment, PMCTA, and molecular autopsy approaches.

We propose a stepwise protocol for the post-mortem investigation of suspected post-COVID cardiovascular deaths, integrating standard pathology with public-health-oriented microbiological assessment, as in Figure 3. Key elements include: (1) systematic clinical and epidemiological history-taking, including documented prior SARS-CoV-2 infection, vaccination status, and known cardiovascular risk factors; (2) external and internal macroscopic examination with PMCTA when available; (3) comprehensive cardiac and pulmonary sampling for histopathology, including conduction-system blocks for SCD cases and segmental pulmonary vascular sampling for PTE cases; (4) tissue-based microbiological workup with RT-PCR and immunohistochemistry for SARS-CoV-2 in cardiac and pulmonary tissue; (5) ante-mortem and post-mortem thrombophilia evaluation where indicated; and (6) molecular autopsy in selected cases of unexplained sudden death. Implementation of such a protocol on a population basis—through coordinated forensic and public health infrastructure—would substantially improve ascertainment of post-COVID cardiovascular mortality. We acknowledge, however, that the routine application of the full protocol described above is unlikely to be feasible across all forensic systems, particularly in low- and middle-income settings where dedicated molecular infrastructure, trained personnel, and sustained funding for advanced post-mortem virological investigations are limited. A tiered approach—in which baseline elements (history-taking, standardized cardiac and pulmonary sampling, basic histopathology) are universally implemented and more resource-intensive components (RT-PCR, IHC, PMCTA, molecular autopsy) are deployed in reference centres or for selected high-yield cases—is therefore likely to be more realistic in routine practice. Ethical considerations relating to molecular autopsy and post-mortem genetic analysis (including informed-consent frameworks for next-of-kin and downstream familial implications of inherited cardiac variants) should also be addressed in any implementation strategy.

## 7. Public Health and Medicolegal Implications

The microbiological and pathological evidence reviewed above has direct implications for public health surveillance, cause-of-death certification, and medicolegal practice in the post-COVID era.

### 7.1. Cause-of-Death Certification and ICD Coding

The certification of cause of death in post-COVID cardiovascular events requires probabilistic causal reasoning that integrates virological, histopathological, epidemiological, and clinical evidence. When post-mortem investigation demonstrates SARS-CoV-2 RNA or antigen in cardiac or pulmonary tissue accompanied by compatible histopathology, the infection may appropriately be cited as the underlying or contributing cause of death, with SCD or PTE recorded as the immediate mechanism. In cases where tissue-based virological evidence is negative but the clinical and epidemiological context—recent symptomatic infection, characteristic histology, exclusion of alternative explanations—strongly supports a causal contribution, a more guarded “contributing factor” formulation is warranted.

Standardized application of ICD-11 codes for post-COVID conditions (codes RA01–RA02 for sequelae of COVID-19) and clear documentation of the basis for causal attribution in the death certificate are essential for surveillance accuracy [80]. It should be emphasized that establishing direct causality between prior SARS-CoV-2 infection and cardiovascular death is inherently challenging, particularly in patients with pre-existing comorbidities, and that such determinations are best framed as probabilistic rather than categorical. International variability in forensic infrastructure, autopsy rates, and death certification practices further complicates uniform implementation of these recommendations; consequently, the proposed framework is intended as guidance to be adapted to local capacity rather than as a uniform global standard. Without such standardization, the population-level burden of post-COVID cardiovascular mortality will remain underestimated and inconsistently reported across jurisdictions.

### 7.2. Medicolegal Considerations in Post-COVID Pulmonary Thromboembolism

Fatal PTE in the post-COVID setting raises specific medicolegal questions. Where death follows hospitalization for COVID-19, the central forensic question is whether thromboprophylaxis was indicated and whether it was administered at appropriate dose and duration. Establishing a malpractice causal link requires demonstration that (i) the standard of care mandated thromboprophylaxis in the patient’s clinical context; (ii) prophylaxis was not administered or was administered at sub-therapeutic doses; and (iii) the deviation more probably than not contributed to the fatal event [81]. Importantly, fatal PTE cannot automatically be attributed to inadequate care: in patients with multiple risk factors, fatal events may occur despite appropriate anticoagulation [82]. The microbiological characterization of post-COVID PTE—including the identification of in situ pulmonary thrombosis driven by primary endothelial pathology—has direct relevance to such causal analyses, by establishing that not all pulmonary thrombi originate from preventable peripheral DVT.

### 7.3. Public Health Surveillance and the Role of Forensic Microbiology

A core argument of this review is that the public health understanding of post-COVID cardiovascular mortality depends on systematic, microbiology-informed forensic investigation. Without standardized post-mortem virological assessment, the contribution of SARS-CoV-2 to out-of-hospital sudden cardiac death and to fatal PTE will remain inadequately characterized, with downstream consequences for surveillance accuracy, public health policy, and the legitimacy of long-COVID research more broadly.

Three priorities follow. First, professional societies in forensic pathology, cardiovascular pathology, and clinical microbiology should collaborate on consensus recommendations for post-mortem investigation of suspected post-COVID cardiovascular deaths, encompassing tissue sampling, RT-PCR/IHC viral detection, thrombophilia evaluation, and molecular autopsy [64]. Second, prospective multicentre forensic registries—ideally linked across jurisdictions and integrated with surveillance infrastructure—are needed to generate population-level evidence on the contribution of SARS-CoV-2 to cardiovascular mortality outside hospital settings. Third, integration of next-generation sequencing into forensic microbiology pipelines would enable variant identification and viral load quantification from FFPE tissue, with applications both for individual cause-of-death analysis and for population-level molecular epidemiology of post-COVID outcomes.

## 8. Limitations

Several limitations of this review warrant acknowledgement. First, the narrative design—as opposed to a formal systematic review—means that study selection was guided by the authors’ judgement of virological, epidemiological, and forensic relevance rather than by a pre-registered, exhaustive search protocol with independent dual-reviewer screening; selection bias and publication bias may therefore have influenced the synthesis. Second, the evidence base is characterized by significant methodological heterogeneity: most autopsy studies are limited to small, single-centre case series, and prospective multicentre autopsy registries specifically designed for post-COVID forensic investigation remain lacking. Third, definitions of “post-COVID” and “long COVID/PASC” vary across studies in terms of required interval since acute infection, symptom burden, and laboratory confirmation, introducing uncertainty when comparing findings; Table 5 summarizes the major case definitions used across key studies and consensus statements, highlighting the heterogeneity in operational criteria. Fourth, the diagnostic criteria for long COVID continue to evolve, and the 2024 National Academies consensus definition has not yet been uniformly adopted in forensic literature; some mechanistic conclusions drawn here may require revision as new data emerge. Fifth, much of the mechanistic evidence for tissue persistence, microclot formation, and complement-driven thromboinflammation derives from studies in living patients with PASC; the extent to which these mechanisms operate in deceased individuals at the time of death requires further direct post-mortem investigation. Sixth, the absence of standardized forensic autopsy registries means that population-level estimates of the contribution of SARS-CoV-2 to SCD and PTE outside hospital settings remain imprecise. Seventh, this review focuses on adult mortality and does not address paediatric post-COVID cardiovascular outcomes, which have a distinct epidemiology and pathophysiology. Eighth, the synthesis may inadvertently over-represent studies supporting the viral persistence hypothesis relative to studies that reported negative or conflicting findings; this potential interpretative imbalance has been mitigated where possible by explicitly noting alternative interpretations within individual sections, but residual bias cannot be excluded. Ninth, much of the autopsy and mechanistic evidence derives from hospitalized or severely affected patients, which may not represent the broader population of individuals with prior SARS-CoV-2 infection, the great majority of whom experienced mild or asymptomatic acute illness. Tenth, the SARS-CoV-2 variant landscape, population-level vaccination coverage, and prior-infection-derived immunity have all evolved substantially across the pandemic, and findings from earlier variants (particularly pre-Omicron) may not extrapolate fully to current epidemiological conditions; recent data on Omicron-era cardiovascular sequelae are still emerging and are not exhaustively covered in this review. Eleventh, the available evidence is predominantly derived from studies conducted in North America and Europe, with limited representation from Asian, African, Latin American, and Oceanian populations; this geographical asymmetry constrains the global generalizability of the synthesized findings, particularly regarding population-level epidemiology, autopsy practice, vaccination coverage, and access to forensic infrastructure, and should be explicitly considered when extrapolating the conclusions of this review to other regions. These limitations underline the urgency of establishing prospective, microbiology-informed forensic registries and harmonized post-mortem protocols as foundational infrastructure for post-COVID public health research.

## 9. Conclusions

The post-COVID era presents a coherent set of challenges that span microbiology, public health, and forensic pathology. Available evidence suggests that SARS-CoV-2 may persist in cardiovascular and other extrapulmonary tissues in at least a subset of patients, that endothelial dysfunction and thromboinflammation can outlast the acute phase of infection in some individuals, and that the pandemic has been accompanied by a measurable population-level excess of cardiovascular and thromboembolic mortality. The degree to which this excess is attributable directly to viral persistence and cardiotropism, as opposed to immune-mediated, coagulation-related, and indirect healthcare-system mechanisms, has not yet been conclusively established, and definitive causal relationships between persistent SARS-CoV-2 reservoirs and sudden cardiac death require further confirmation in prospective longitudinal studies and standardized autopsy registries.

Sudden cardiac death and fatal pulmonary thromboembolism are the two outcomes at which these mechanisms most prominently converge in forensic practice. Their proper investigation requires integration of standard autopsy techniques with virological tools—tissue-based RT-PCR, immunohistochemistry for SARS-CoV-2 antigens, and molecular autopsy—that have historically belonged to the clinical microbiology and infectious disease laboratory rather than to the forensic suite. The development of consensus protocols, prospective multicentre registries, prospective longitudinal cohort studies of post-COVID cardiovascular outcomes, and mechanistic validation studies of viral persistence in human cardiac tissue will be essential for clarifying the true contribution of SARS-CoV-2 to long-term cardiovascular pathology, and for ensuring that cause-of-death certification and medicolegal practice in the years ahead reflect the best available microbiological evidence.

A microbiology-informed, public-health-oriented approach to forensic investigation is therefore not merely a technical refinement but a structural prerequisite for understanding, surveilling, and ultimately mitigating the long-term cardiovascular consequences of the SARS-CoV-2 pandemic.

## Figures and Tables

**Table 1:** Summary of literature search strategy and study selection.

Step	Details
Databases searched	PubMed (MEDLINE), Scopus, Web of Science Core Collection
Search period	1 January 2020 to 31 December 2025
Search blocks	(a) Virological (SARS-CoV-2, ACE2, tropism, persistence, reservoir); (b) Immunopathological (endothelial dysfunction, NETosis, thromboinflammation, complement); (c) Clinical/pathological outcomes (SCD, myocarditis, PE, VTE, DVT, long COVID, PASC); (d) Public health and forensic (excess mortality, autopsy, cause of death, medicolegal)
Records identified (*n*)	1837 (PubMed: 974; Scopus: 558; WoS: 305)
Duplicates removed	412
Records screened (title/abstract)	1425
Full-text records assessed for eligibility	198
Exclusion criteria	Conference abstracts without full text; non-peer-reviewed preprints; paediatric-only cohorts; studies addressing acute COVID-19 only without post-acute, autopsy, or surveillance dimension; non-English publications; case reports without histopathological or molecular data
Studies included in final synthesis	78 (original articles, autopsy series, mechanistic studies, systematic and narrative reviews, epidemiological surveillance analyses, position statements)
Design note	Narrative (non-systematic) review. No PRISMA-guided dual-reviewer screening or formal risk-of-bias assessment was performed. Selection guided by virological, epidemiological, and forensic relevance and methodological quality.

PASC = post-acute sequelae of SARS-CoV-2; PE = pulmonary embolism; SCD = sudden cardiac death; VTE = venous thromboembolism; WoS = Web of Science.

**Table 2:** Comparative summary of major population-level studies of post-COVID cardiovascular and thromboembolic risk: hazard ratios, follow-up durations, and key outcomes.

Study (Year, Country)	Population (*n*)	Outcome	HR/aHR (95% CI)	Follow-Up	Vaccination Effect
Xie et al., 2022 (USA, VA cohort) [59]	153,760 COVID-19 cases vs. 5,637,647 contemporary controls	Composite cardiovascular outcomes (12 months)	HR 1.63 (1.59–1.68) for any CVD	12 months post-acute	Risk graded by acute severity; non-hospitalized included
Bowe et al., 2023 (USA, VA cohort) [16]	138,818 COVID-19 cases vs. 5,985,227 controls	Cardiovascular sequelae at 2 years	Sustained elevated HRs across CVD outcomes	2 years post-acute	Risk attenuated but persistent at 2 years
Han et al., 2023 (USA) [11]	National vital statistics	Excess cardiovascular deaths	90,160 excess CVD deaths (4.9% above expected)	March 2020–March 2022	Coincident with pandemic waves
Woodruff et al., 2024 (USA, CDC) [3]	National vital statistics	Excess cardiovascular mortality	228,524 excess CVD deaths (9.0%, 95% CI 7.8–10.3)	2020–2022	Greatest excess in adults 35–54 years
Kim et al., 2024 (South Korea) [12]	1,601,835 COVID-19 cases vs. 14,011,285 controls	Pulmonary embolism (PE)	aHR 6.25 (3.67–10.66) unvaccinated; 1.48 (1.15–1.88) vaccinated	Up to 12 months	Substantial attenuation in vaccinated
Kim et al., 2024 (South Korea) [12]	as above	Deep vein thrombosis (DVT)	aHR 3.05 (1.75–5.29) unvaccinated	Up to 12 months	Attenuated in vaccinated
Sjöland et al., 2023 (Sweden) [13]	Population-based cohort	PE in 60–180-day window	Elevated HRs, attenuating beyond 180 d	60 days to >180 days	Pre-Omicron era predominantly
Wasfy et al., 2025 (USA) [58]	National vital statistics	Age-adjusted cardiac mortality	Above pre-pandemic projections through 2024	Through 2024	Persistent in elderly subgroups
Al-Aly et al., 2022 (USA, VA cohort) [15]	Vaccinated breakthrough infections vs. uninfected	Long COVID (incl. cardiovascular)	Attenuated risk vs. unvaccinated; not eliminated	6 months post-acute	Vaccination partially protective

aHR = adjusted hazard ratio; CVD = cardiovascular disease; CI = confidence interval; HR = hazard ratio; PE = pulmonary embolism; DVT = deep vein thrombosis; VA = Veterans Affairs.

**Table 3:** Selected autopsy series of COVID-19-related cardiac death: pathological findings and tissue-based viral detection.

Study (Year)	*n*	Lymphocytic Infiltrate/Myocarditis	Microvascular Thrombosis/Contraction-Band Necrosis	Other Histological Findings	SARS-CoV-2 in Cardiac Tissue
Lindner et al., 2020 (*JAMA Cardiol.*) [36]	39	Mononuclear infiltrates without Dallas-criteria myocarditis	Not specifically reported	Pro-inflammatory cytokine signature in high viral-load cases	RT-PCR positive in 24/39 (61.5%); >1000 copies/μg RNA in 16/39 (41.0%)
Basso et al., 2020 (*Eur. Heart J.*) [37]	21	Myocarditis (Dallas criteria) in 3/21 (14.3%); focal lymphocytic infiltrates in additional cases	Microvascular thrombi present in subset	Pericarditis in 4/21; macrophage-predominant infiltrates	RT-PCR/IHC positive in subset, frequently in interstitial macrophages
Halushka & Vander Heide, 2021 (*Cardiovasc. Pathol.*) [38]	277 (literature review)	Histological myocarditis in ~7.2%; functionally significant in <2%	Variable across reports	Heterogeneous findings; non-specific changes common	Variable detection; not consistently reported
Che et al., 2025 (*J. Adv. Res.*) [56]	5 (endomyocardial biopsy)	Inflammatory infiltrates documented	Not reported	Extensive mitochondrial vacuolation, myofilament degradation, lipofuscin accumulation (electron microscopy)	Not directly assessed
Kyuno et al., 2023 (*Heliyon*) [67]	2	No inflammatory infiltrates on H&E	Multivacuolation; loss of rhabdomeres	Decreased special-stain intensity	Not performed

H&E = haematoxylin and eosin; IHC = immunohistochemistry; RT-PCR = reverse transcription polymerase chain reaction.

**Table 4:** Risk factors for fatal pulmonary thromboembolism in the post-COVID setting compared with the general population.

Risk Factor	General Population (Non-COVID PTE)	Post-COVID PTE	Comment
Advanced age (>65 years)	Strong risk factor	Strong; amplified by comorbidities	Elderly retain excess COVID-related cardiovascular mortality through 2024 [58]
Immobilization/hospitalization	Major	Major; extended by post-COVID fatigue	Venous stasis may persist weeks after hospital discharge
Prior venous thromboembolism	Major independent factor	Major independent factor	Should be sought in pre-mortem clinical records
Obesity (BMI > 30)	Independent factor	Independent; amplified by COVID severity	Increases DVT-source burden
Active malignancy	Well-established	Additive risk with COVID	Shared hypercoagulable mechanisms
SARS-CoV-2 endotheliitis	Not applicable	Central, COVID-specific pathway; persists in long COVID	Distinguishes post-COVID PTE from classical VTE; assessable by IHC at autopsy
Antiphospholipid antibodies	Classical acquired thrombophilia	De novo induction by SARS-CoV-2; persistence variable	Ante-mortem serology where available is informative
NETosis/platelet hyperactivation	Minimal role in non-infectious PTE	Central mechanism; persists in PASC	COVID-specific mechanism; biomarker research ongoing
Inherited thrombophilia (FVL, PT G20210A)	Well-established genetic risk	Amplifies post-COVID thromboembolic risk	Post-mortem molecular genetic testing indicated in selected cases
Absence of DVT source at autopsy	~30% of PTE cases lack confirmed DVT	Higher proportion; in situ pulmonary thrombosis common	Suggests primary pulmonary microvascular pathology

BMI = body mass index; DVT = deep vein thrombosis; FVL = Factor V Leiden; IHC = immunohistochemistry; PASC = post-acute sequelae of SARS-CoV-2; PT = prothrombin gene variant; PTE = pulmonary thromboembolism; VTE = venous thromboembolism.

**Table 5:** Comparison of major case definitions of long COVID/post-acute sequelae of SARS-CoV-2 infection (PASC) used across key studies and consensus statements.

Source/Definition	Minimum Interval Since Acute Infection	Key Clinical Criteria	Notes for Forensic/Epidemiological Use
WHO clinical case definition (October 2021)	Usually 3 months from onset of probable/confirmed COVID-19	Symptoms lasting ≥2 months that cannot be explained by an alternative diagnosis; impact on functioning	Most widely used in international clinical research; symptom-focused
NICE/RCGP (UK, updated)	≥4 weeks (ongoing symptomatic COVID-19: 4–12 weeks; post-COVID-19 syndrome: ≥12 weeks)	Signs/symptoms developed during or after COVID-19, continuing ≥12 weeks, not explained by another diagnosis	Two-stage definition; shorter post-acute window
CDC/US HHS (working definition)	≥4 weeks after initial SARS-CoV-2 infection	Broad spectrum of new, returning, or ongoing health problems	Operationally inclusive; used in US surveillance
National Academies of Sciences, Engineering, and Medicine (NASEM) 2024 consensus	≥3 months after initial SARS-CoV-2 infection	Chronic condition presenting with one or more symptoms; may be continuous, relapsing-remitting, or progressive	Designed for clinical, research, and policy use; latest consensus, not yet uniformly adopted in forensic literature
RECOVER (NIH research definition)	≥6 months in most analyses	Symptom-cluster phenotyping; data-driven scoring	Research instrument; not directly applicable to certification
Most autopsy/forensic studies cited in this review	Variable, often unspecified or operationally defined	Documented prior SARS-CoV-2 infection plus death attributable to compatible cardiovascular event	Heterogeneous; limits direct comparison across forensic cohorts

CDC = Centers for Disease Control and Prevention; HHS = Health and Human Services; NASEM = National Academies of Sciences, Engineering, and Medicine; NICE = National Institute for Health and Care Excellence; RCGP = Royal College of General Practitioners; RECOVER = Researching COVID to Enhance Recovery; WHO = World Health Organization.

## Data Availability

No new data were created or analyzed in this study. Data sharing is not applicable to this article.

## Abbreviations

The following abbreviations are used in this manuscript:

ACE2	Angiotensin-converting enzyme 2
aHR	Adjusted hazard ratio
aPL	Antiphospholipid antibodies
BMI	Body mass index
CBN	Contraction band necrosis
COVID-19	Coronavirus disease 2019
DVT	Deep vein thrombosis
FFPE	Formalin-fixed paraffin-embedded
FVL	Factor V Leiden
H&E	Haematoxylin and eosin
hPSC-CM	Human pluripotent stem cell-derived cardiomyocyte
ICD	International Classification of Diseases
IHC	Immunohistochemistry
MeSH	Medical Subject Headings
NET	Neutrophil extracellular trap
OHCA	Out-of-hospital cardiac arrest
PASC	Post-acute sequelae of SARS-CoV-2 infection
PE	Pulmonary embolism
PMCTA	Post-mortem computed tomography pulmonary angiography
PRISMA	Preferred Reporting Items for Systematic Reviews and Meta-Analyses
PTE	Pulmonary thromboembolism
RT-PCR	Reverse transcription polymerase chain reaction
SARS-CoV-2	Severe acute respiratory syndrome coronavirus 2
SCD	Sudden cardiac death
TMPRSS2	Transmembrane protease serine 2
VTE	Venous thromboembolism
VWF	Von Willebrand factor
WoS	Web of Science

## References

[1] World Health Organization (2025). COVID-19 Dashboard.

[2] Davis H.E., McCorkell L., Vogel J.M., Topol E.J. (2023). Long COVID: Major findings, mechanisms and recommendations. Nat. Rev. Microbiol..

[3] Woodruff R.C., Tong X., Khan S.S., Shah N.S., Jackson S.L., Loustalot F., Vaughan A.S. (2024). Trends in cardiovascular disease mortality rates and excess deaths, 2010–2022. Am. J. Prev. Med..

[4] Hoffmann M., Kleine-Weber H., Schroeder S., Krüger N., Herrler T., Erichsen S., Schiergens T.S., Herrler G., Wu N.H., Nitsche A. (2020). SARS-CoV-2 cell entry depends on ACE2 and TMPRSS2 and is blocked by a clinically proven protease inhibitor. Cell.

[5] Litviňuková M., Talavera-López C., Maatz H., Reichart D., Worth C.L., Lindberg E.L., Kanda M., Polanski K., Heinig M., Lee M. (2020). Cells of the adult human heart. Nature.

[6] Chen C., Wang J., Liu Y.-M., Hu J. (2023). Single-cell analysis of adult human heart across healthy and cardiovascular disease patients reveals the cellular landscape underlying SARS-CoV-2 invasion of myocardial tissue through ACE2. J. Transl. Med..

[7] Stein S.R., Ramelli S.C., Grazioli A., Chung J.-Y., Singh M., Yinda C.K., Winkler C.W., Sun J., Dickey J.M., Ylaya K. (2022). SARS-CoV-2 infection and persistence in the human body and brain at autopsy. Nature.

[8] Proal A.D., VanElzakker M.B., Aleman S., Bach K., Boribong B.P., Buggert M., Cherry S., Chertow D.S., Davies H.E., Dupont C.L. (2023). SARS-CoV-2 reservoir in post-acute sequelae of COVID-19 (PASC). Nat. Immunol..

[9] Nicolai L., Kaiser R., Stark K. (2023). Thromboinflammation in long COVID—The elusive key to postinfection sequelae?. J. Thromb. Haemost..

[10] Pretorius E., Venter C., Bhatt D.L., Kell D.B. (2021). Persistent clotting protein pathology in long COVID/post-acute sequelae of COVID-19 is accompanied by increased levels of antiplasmin. Cardiovasc. Diabetol..

[11] Han L., Zhao S., Li S., Gu S., Deng X., Yang L., Ran J. (2023). Excess cardiovascular mortality across multiple COVID-19 waves in the United States from March 2020 to March 2022. Nat. Cardiovasc. Res..

[12] Kim H.J., Jeong S., Song J., Park S.J., Park Y.J., Oh Y.H., Jung J., Park S.M. (2024). Risk of pulmonary embolism and deep vein thrombosis following COVID-19: A nationwide cohort study. MedComm.

[13] Sjöland H., Lindgren M., Toska T., Hansson P.O., Glise Sandblad K., Alex C., Björck L., Cronie O., Björk J., Lundberg C.E. (2023). Pulmonary embolism and deep venous thrombosis after COVID-19: Long-term risk in a population-based cohort study. Res. Pract. Thromb. Haemost..

[14] Polack F.P., Thomas S.J., Kitchin N., Absalon J., Gurtman A., Lockhart S., Perez J.L., Pérez Marc G., Moreira E.D., Zerbini C. (2020). Safety and efficacy of the BNT162b2 mRNA COVID-19 vaccine. N. Engl. J. Med..

[15] Al-Aly Z., Bowe B., Xie Y. (2022). Long COVID after breakthrough SARS-CoV-2 infection. Nat. Med..

[16] Bowe B., Xie Y., Al-Aly Z. (2023). Postacute sequelae of COVID-19 at 2 years. Nat. Med..

[17] Iwasaki M., Saito J., Zhao H., Sakamoto A., Hirota K., Ma D. (2021). Inflammation triggered by SARS-CoV-2 and ACE2 augment drives multiple organ failure of severe COVID-19: Molecular mechanisms and implications. Inflammation.

[18] Maiese A., Manetti A.C., La Russa R., Di Paolo M., Turillazzi E., Frati P., Fineschi V. (2021). Autopsy findings in COVID-19-related deaths: A literature review. Forensic Sci. Med. Pathol..

[19] Lisman D., Zielińska G., Drath J., Łaszczewska A., Savochka I., Parafiniuk M., Ossowski A. (2023). Molecular diagnosis of COVID-19 sudden and unexplained deaths: The insidious face of the pandemic. Diagnostics.

[20] Sungnak W., Huang N., Bécavin C., Berg M., Queen R., Litvinukova M., Talavera-López C., Maatz H., Reichart D., Sampaziotis F. (2020). SARS-CoV-2 entry factors are highly expressed in nasal epithelial cells together with innate immune genes. Nat. Med..

[21] Pérez-Bermejo J.A., Kang S., Rockwood S.J., Simoneau C.R., Joy D.A., Silva A.C., Ramadoss G.N., Flanigan W.R., Fozouni P., Li H. (2021). SARS-CoV-2 infection of human iPSC-derived cardiac cells reflects cytopathic features in hearts of patients with COVID-19. Sci. Transl. Med..

[22] Marchiano S., Hsiang T.-Y., Khanna A., Higashi T., Whitmore L.S., Bargehr J., Davaapil H., Chang J., Smith E., Ong L.P. (2021). SARS-CoV-2 infects human pluripotent stem cell-derived cardiomyocytes, impairing electrical and mechanical function. Stem Cell Rep..

[23] Zhang J., He M., Xie Q., Su A., Yang K., Liu L., Wang Y. (2022). Predicting in vitro and in vivo anti-SARS-CoV-2 activities of antivirals by intracellular bioavailability and biochemical activity. ACS Omega.

[24] Varga Z., Flammer A.J., Steiger P., Haberecker M., Andermatt R., Zinkernagel A.S., Mehra M.R., Schuepbach R.A., Ruschitzka F., Moch H. (2020). Endothelial cell infection and endotheliitis in COVID-19. Lancet.

[25] Ackermann M., Verleden S.E., Kuehnel M., Haverich A., Welte T., Laenger F., Vanstapel A., Werlein C., Stark H., Tzankov A. (2020). Pulmonary vascular endothelialitis, thrombosis, and angiogenesis in COVID-19. N. Engl. J. Med..

[26] Dmytrenko O., Lavine K.J. (2022). Cardiovascular tropism and sequelae of SARS-CoV-2 infection. Viruses.

[27] Swank Z., Senussi Y., Manickas-Hill Z., Yu X.G., Li J.Z., Alter G., Walt D.R. (2023). Persistent circulating severe acute respiratory syndrome coronavirus 2 spike is associated with post-acute coronavirus disease 2019 sequelae. Clin. Infect. Dis..

[28] Schultheiß C., Willscher E., Paschold L., Gottschick C., Klee B., Henkes S.S., Bosurgi L., Dutzmann J., Sedding D., Frese T. (2022). The IL-1β, IL-6, and TNF cytokine triad is associated with post-acute sequelae of COVID-19. Cell Rep. Med..

[29] Tejerina F., Catalan P., Rodriguez-Grande C., Adan J., Rodriguez-Gonzalez C., Muñoz P., Aldamiz T., Diez C., Perez L., Fanciulli C. (2022). Post-COVID-19 syndrome: SARS-CoV-2 RNA detection in plasma, stool, and urine in patients with persistent symptoms after COVID-19. BMC Infect. Dis..

[30] Zollner A., Koch R., Jukic A., Pfister A., Meyer M., Rössler A., Kimpel J., Adolph T.E., Tilg H. (2022). Postacute COVID-19 is characterized by gut viral antigen persistence in inflammatory bowel diseases. Gastroenterology.

[31] Rong Z., Mai H., Ebert G., Kapoor S., Puelles V.G., Czogalla J., Hu S., Su J., Prtvar D., Singh I. (2024). Persistence of spike protein at the skull-meninges-brain axis may contribute to the neurological sequelae of COVID-19. Cell Host Microbe.

[32] Klein J., Wood J., Jaycox J.R., Dhodapkar R.M., Lu P., Gehlhausen J.R., Tabachnikova A., Greene K., Tabacof L., Malik A.A. (2023). Distinguishing features of long COVID identified through immune profiling. Nature.

[33] Zheng Y., Zhao J., Li J., Guo Z., Sheng J., Ye X., Jin G., Wang C., Chai W., Yan J. (2021). SARS-CoV-2 spike protein causes blood coagulation and thrombosis by competitive binding to heparan sulfate. Int. J. Biol. Macromol..

[34] Kell D.B., Laubscher G.J., Pretorius E. (2022). A central role for amyloid fibrin microclots in long COVID/PASC: Origins and therapeutic implications. Biochem. J..

[35] Zhang Y., Bharathi V., Dokoshi T., de Anda J., Ursery L.T., Kulkarni N.N., Nakamura Y., Chen J., Luo E.W.C., Wang L. (2024). Viral afterlife: SARS-CoV-2 as a reservoir of immunomimetic peptides that reassemble into proinflammatory supramolecular complexes. Proc. Natl. Acad. Sci. USA.

[36] Lindner D., Fitzek A., Bräuninger H., Aleshcheva G., Edler C., Meissner K., Scherschel K., Kirchhof P., Escher F., Schultheiss H.-P. (2020). Association of cardiac infection with SARS-CoV-2 in confirmed COVID-19 autopsy cases. JAMA Cardiol..

[37] Basso C., Leone O., Rizzo S., De Gaspari M., van der Wal A.C., Aubry M.-C., Bois M.C., Lin P.T., Maleszewski J.J., Stone J.R. (2020). Pathological features of COVID-19-associated myocardial injury: A multicentre cardiovascular pathology study. Eur. Heart J..

[38] Halushka M.K., Vander Heide R.S. (2021). Myocarditis is rare in COVID-19 autopsies: Cardiovascular findings across 277 postmortem examinations. Cardiovasc. Pathol..

[39] Caforio A.L.P., Pankuweit S., Arbustini E., Basso C., Gimeno-Blanes J., Felix S.B., Fu M., Heliö T., Heymans S., Jahns R. (2013). Current state of knowledge on aetiology, diagnosis, management, and therapy of myocarditis: A position statement of the European Society of Cardiology Working Group on Myocardial and Pericardial Diseases. Eur. Heart J..

[40] Falleti J., Orabona P., Municinò M., Castellaro G., Fusco G., Mansueto G. (2024). An update on myocarditis in forensic pathology. Diagnostics.

[41] Ambrosino P., Calcaterra I., Molino A., Moretta P., Lupoli R., Spedicato G.A., Papa A., Motta A., Maniscalco M., Di Minno M.N.D. (2021). Persistent endothelial dysfunction in post-acute COVID-19 syndrome: A case-control study. Biomedicines.

[42] Evans P.C., Rainger G.E., Mason J.C., Guzik T.J., Osto E., Stamataki Z., Neil D., Hoefer I.E., Fragiadaki M., Waltenberger J. (2020). Endothelial dysfunction in COVID-19: A position paper of the ESC Working Group for Atherosclerosis and Vascular Biology, and the ESC Council of Basic Cardiovascular Science. Cardiovasc. Res..

[43] Charfeddine S., Ibn Hadj Amor H., Jdidi J., Torjmen S., Kraiem S., Hammami R., Bahloul A., Kallel N., Moussa N., Touil I. (2021). Long COVID-19 syndrome: Is it related to microcirculation and endothelial dysfunction?. Front. Cardiovasc. Med..

[44] Thomas D., Noishiki C., Gaddam S., Wu D., Manhas A., Liu Y., Tripathi D., Kathale N., Adkar S.S., Garhyan J. (2024). CCL2-mediated endothelial injury drives cardiac dysfunction in long COVID. Nat. Cardiovasc. Res..

[45] Kang H., Yan G., Lin X., Tian Y., Yin J., Liu J., Deng Z., Guo J., Lu J., Lin X. (2025). The substructure of the endothelial glycocalyx in rat aorta and its interaction with the low-density lipoproteins. Am. J. Pathol..

[46] Zuo Y., Yalavarthi S., Shi H., Gockman K., Zuo M., Madison J.A., Blair C., Weber A., Barnes B.J., Egeblad M. (2020). Neutrophil extracellular traps in COVID-19. JCI Insight.

[47] Middleton E.A., He X.-Y., Denorme F., Campbell R.A., Ng D., Salvatore S.P., Mostyka M., Baxter-Stoltzfus A., Borczuk A.C., Loda M. (2020). Neutrophil extracellular traps contribute to immunothrombosis in COVID-19 acute respiratory distress syndrome. Blood.

[48] Aggarwal A., Singh T.K., Pham M., Godwin M., Chen R., McIntyre T.M., Scalise A., Chung M.K., Jennings C., Ali M. (2024). Dysregulated platelet function in patients with postacute sequelae of COVID-19. Vasc. Med..

[49] Manne B.K., Denorme F., Middleton E.A., Portier I., Rowley J.W., Stubben C., Petrey A.C., Tolley N.D., Guo L., Cody M. (2020). Platelet gene expression and function in patients with COVID-19. Blood.

[50] Liu W., Li G., Shi J., Gao Y., Fang P., Zhao Y., Zhong F., Guo X., Lyu Y., Da X. (2025). NR4A1 acts as a novel regulator of platelet activation and thrombus formation. Circ. Res..

[51] Magro C., Mulvey J.J., Berlin D., Nuovo G., Salvatore S., Harp J., Baxter-Stoltzfus A., Laurence J. (2020). Complement associated microvascular injury and thrombosis in the pathogenesis of severe COVID-19 infection: A report of five cases. Transl. Res..

[52] Pretorius E., Venter C., Laubscher G.J., Kotze M.J., Oladejo S.O., Watson L.R., Rajaratnam K., Watson B.W., Kell D.B. (2022). Prevalence of symptoms, comorbidities, fibrin amyloid microclots and platelet pathology in individuals with long COVID/PASC. Cardiovasc. Diabetol..

[53] Panigada M., Bottino N., Tagliabue P., Grasselli G., Novembrino C., Iapichino G., Pesenti A., Ghilardi G., Gossi U., Casella G. (2020). Hypercoagulability of COVID-19 patients in intensive care unit: A report of thromboelastography findings and other parameters of hemostasis. J. Thromb. Haemost..

[54] Yong S.J. (2021). Long COVID or post-COVID-19 syndrome: Putative pathophysiology, risk factors, and treatments. Infect. Dis..

[55] Zhang Y., Xiao M., Zhang S., Xia P., Cao W., Jiang W., Chen H., Ding X., Zhao H., Zhang H. (2020). Coagulopathy and antiphospholipid antibodies in patients with COVID-19. N. Engl. J. Med..

[56] Che W., Guo S., Wang Y., Wan X., Tan B., Li H., Alifu J., Zhu M., Chen Z., Li P. (2025). SARS-CoV-2 damages cardiomyocyte mitochondria and implicates long COVID-associated cardiovascular manifestations. J. Adv. Res..

[57] Shang C., Liu Z., Zhu Y., Lu J., Ge C., Zhang C., Li N., Jin N., Li Y., Tian M. (2022). SARS-CoV-2 causes mitochondrial dysfunction and mitophagy impairment. Front. Microbiol..

[58] Wasfy J.H., Lin Y., Price M., Newhouse J.P., Blacker D., Hsu J. (2025). Postpandemic cardiac mortality rates. JAMA Netw. Open.

[59] Xie Y., Xu E., Bowe B., Al-Aly Z. (2022). Long-term cardiovascular outcomes of COVID-19. Nat. Med..

[60] Christensen M.R., Larsen A.B., Boel L.W.T. (2025). Autopsy characteristics of deaths due to pulmonary thromboembolism in northern and western Denmark: A 10-year retrospective study. Forensic Sci. Med. Pathol..

[61] Marijon E., Karam N., Jost D., Perrot D., Frattini B., Derkenne C., Sharifzadehgan A., Waldmann V., Beganton F., Narayanan K. (2020). Out-of-hospital cardiac arrest during the COVID-19 pandemic in Paris, France: A population-based, observational study. Lancet Public Health.

[62] Song S., Guo C., Wu R., Zhao H., Li Q., Dou J.-H., Guo F.-S., Wei J. (2024). Impact of the COVID-19 pandemic on cardiovascular mortality and contrast analysis within subgroups. Front. Cardiovasc. Med..

[63] Myerburg R.J., Castellanos A., Zipes D.P., Libby P., Bonow R.O., Mann D.L., Tomaselli G.F. (2019). Cardiac arrest and sudden cardiac death. Braunwald’s Heart Disease: A Textbook of Cardiovascular Medicine.

[64] Lynge T.H., Albert C.M., Basso C., Garcia R., Krahn A.D., Semsarian C., Sheppard M.N., Behr E.R., Tfelt-Hansen J. (2024). Autopsy examination in sudden cardiac death: A current perspective on behalf of the Association for European Cardiovascular Pathology. Europace.

[65] Basso C., Aguilera B., Banner J., Cohle S., d’Amati G., de Gouveia R.H., di Gioia C., Fabre A., Gallagher P.J., Leone O. (2017). Guidelines for autopsy investigation of sudden cardiac death: 2017 update from the Association for European Cardiovascular Pathology. Virchows Arch..

[66] Pellegrini D., Kawakami R., Guagliumi G., Sakamoto A., Kawai K., Gianatti A., Nasr A., Kutys R., Guo L., Cornelissen A. (2021). Microthrombi as a major cause of cardiac injury in COVID-19: A pathologic study. Circulation.

[67] Kyuno D., Tateno M., Ono Y., Magara K., Takasawa K., Takasawa A., Osanai M. (2023). Common pathological findings in the heart in COVID-19-related sudden death cases: An autopsy case series. Heliyon.

[68] Paruchuri S.S.H., Farwa U.E., Jabeen S., Pamecha S., Shan Z., Parekh R., Lakkimsetti M., Alamin E., Sharma V., Haider S. (2023). Myocarditis and myocardial injury in long COVID syndrome: A comprehensive review of the literature. Cureus.

[69] Bois M.C., Boire N.A., Layman A.J., Aubry M.-C., Alexander M.P., Roden A.C., Hagen C.E., Quinton R.A., Larsen C.P., Erben Y. (2021). COVID-19-associated nonocclusive fibrin microthrombi in the heart. Circulation.

[70] Oyarzun A., Parsons S., Bassed R. (2023). Myocarditis in the forensic setting. Cardiovasc. Pathol..

[71] Chen Z., Li J., Li T., Fan T., Meng C., Li C., Kang J., Chai L., Hao Y., Tang Y. (2022). A CRISPR/Cas12a-empowered surface plasmon resonance platform for rapid and specific diagnosis of the Omicron variant of SARS-CoV-2. Natl. Sci. Rev..

[72] Campuzano O., Sarquella-Brugada G., Fernandez-Falgueras A., Coll M., Iglesias A., Ferrer-Costa C., Cesar S., Arbelo E., Brugada J., Brugada R. (2023). Molecular autopsy: Twenty years of post-mortem diagnosis in sudden cardiac death. Front. Med..

[73] Doyle L.W., Tsokos M. (2005). Medicolegal evaluation of fatal pulmonary thromboembolism. Forensic Pathology Reviews.

[74] Molina D.K., Farley N.J., DiMaio V.J.M. (2025). Cadaver clots: A systematic review of the literature. Forensic Sci. Med. Pathol..

[75] Garland J., Tse R., Bhatt S., Ramachandran A., Klein T., Langlois N.E.I. (2014). Can forensic pathologists diagnose pulmonary thromboembolism on postmortem computed tomography pulmonary angiography?. Am. J. Forensic Med. Pathol..

[76] Lax S.F., Skok K., Zechner P., Kessler H.H., Kaufmann N., Koelblinger C., Vander K., Bargfrieder U., Trauner M. (2020). Pulmonary arterial thrombosis in COVID-19 with fatal outcome: Results from a prospective, single-center, clinicopathologic case series. Ann. Intern. Med..

[77] Bryce C., Grimes Z., Pujadas E., Ahuja S., Beasley M.B., Albrecht R., Hernandez T., Stock A., Zhao Z., AlRasheed M.R. (2021). Pathophysiology of SARS-CoV-2: The Mount Sinai COVID-19 autopsy experience. Mod. Pathol..

[78] Edler C., Schröder A.S., Aepfelbacher M., Fitzek A., Heinemann A., Heinrich F., Klein A., Langenwalder F., Lütgehetmann M., Meißner K. (2020). Dying with SARS-CoV-2 infection—An autopsy study of the first consecutive 80 cases in Hamburg, Germany. Int. J. Leg. Med..

[79] Wichmann D., Sperhake J.-P., Lütgehetmann M., Steurer S., Edler C., Heinemann A., Heinrich F., Mushumba H., Kniep I., Schröder A.S. (2020). Autopsy findings and venous thromboembolism in patients with COVID-19: A prospective cohort study. Ann. Intern. Med..

[80] World Health Organization (2024). ICD-11 for Mortality and Morbidity Statistics: Coding for COVID-19 and Post-COVID-19 Condition.

[81] Mobilia F., Casali M.B., Gallieni M., Genovese U.R. (2014). Lethal pulmonary thromboembolism: An autopsy-based study on a rare but legally relevant event. Med. Sci. Law.

[82] Ro A., Kageyama N., Tanifuji T., Fukunaga T. (2008). Pulmonary thromboembolism: Overview and update from medicolegal aspects. Leg. Med..

